# Cyclodextrin-assisted surface-enhanced Raman spectroscopy: a critical review

**DOI:** 10.1007/s00216-021-03704-x

**Published:** 2021-10-11

**Authors:** Natalia E. Markina, Dana Cialla-May, Alexey V. Markin

**Affiliations:** 1 Institute of Chemistry, Saratov State University, Astrakhanskaya 83, 410012 Saratov, Russia; 2 Leibniz Institute of Photonic Technology, Member of the Leibniz Research Alliance, “Leibniz Health Technologies”, Albert-Einstein-Straße 9, 07745 Jena, Germany; 3 Institute of Physical Chemistry and Abbe Center of Photonics, Friedrich Schiller University Jena, Helmholtzweg 4, 07743 Jena, Germany; 4 InfectoGnostics Research Campus Jena, Center for Applied Research, Friedrich-Schiller-University, Philosophenweg 7, 07743 Jena, Germany

**Keywords:** Cyclodextrins, SERS, Inclusion complexes, Molecular recognition, Stereoselectivity, Sorption

## Abstract

Numerous approaches have been proposed to overcome the intrinsically low selectivity of surface-enhanced Raman spectroscopy (SERS), and the modification of SERS substrates with diverse recognition molecules is one of such approaches. In contrast to the use of antibodies, aptamers, and molecularly imprinted polymers, application of cyclodextrins (CDs) is still developing with less than 100 papers since 1993. Therefore, the main goal of this review is the critical analysis of all available papers on the use of CDs in SERS analysis, including physicochemical studies of CD complexation and the effect of CD presence on the Raman enhancement. The results of the review reveal that there is controversial information about CD efficiency and further experimental investigations have to be done in order to estimate the real potential of CDs in SERS-based analysis.

**Graphical abstract:**

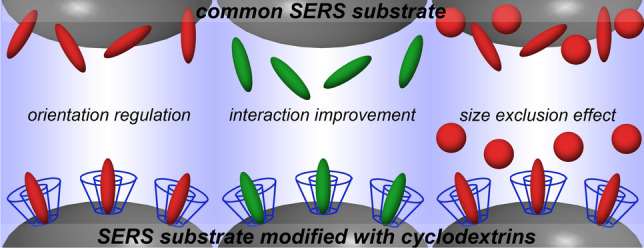

## Introduction

Surface-enhanced Raman spectroscopy (SERS) is a vibrational spectroscopy that utilizes plasmonic nanostructures (SERS substrates) to enhance weak Raman signal to several orders of magnitude (generally 10^4^–10^8^ times). The mechanism of SERS and its main advantages for analytical chemistry have been comprehensively described and analyzed in the review papers [1, 2]. However, SERS also has some limitations and the most important one is intrinsically low selectivity because the Raman signal of any molecule adsorbed on the surface of SERS substrate will be enhanced.

Low selectivity significantly restricts application of SERS for analysis of complex mixtures such as body fluids, and several approaches have been proposed to overcome this limitation. The simplest one is the adjustment of pH, ionic strength, and/or dielectric constant of the analyzed solution to maximize interaction of analyte molecules with SERS substrate and minimize such interaction for admixtures [3–5]. The next approach implies preliminary separation of the analyte by chromatographic separation or by solid-phase/liquid-liquid extraction [6–8]. The last approach is based on modification of SERS substrates by various recognition elements, e.g., by antibodies, aptamers, and molecularly imprinted polymers. Specific cases and general overviews of this approach have been already analyzed in several reviews dedicated to molecular trapping [9], molecularly imprinted polymers [10], and SERS nanotags (label-based SERS analysis) [11].

**Fig. 1:**
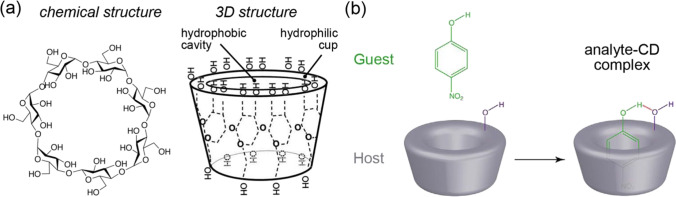
**a** An example of structure of native cyclodextrin (CD) consisted of 7 linked glucopyranose subunits (β-CD). **b** Schematic illustration of an inclusion complex with CD, adapted with permission from ref. [14]

Besides the listed recognition elements, cyclodextrins (CDs) have also been proposed for modification of SERS substrates to improve selectivity and, consequently, reliability of SERS-based analysis. CDs are a family of cyclic oligosaccharides composed of linked glucopyranose subunits (Fig. 1a) which form structures with a cavity suitable for formation of inclusion (“host-guest”) complexes (Fig. 1b) [12]. This structure enables the use of CDs as sorbents and recognition elements, and chemical modification of one or both rims enables additional improvement of selectivity and sorption capacity. In the case of hydrophobic guests, the formation of such complexes enables to improve solubility of the guests in water. Therefore, the inclusion complexes with CDs have found diverse applications for solubilization and stabilization of bioactive molecules in food science [13], pharmacy [12], and water purification [14]. In chemical analysis, CDs are also used for separation and chiral recognition [15–17]. Besides improving selectivity, the use of CDs in SERS is attractive due to reasonable cost-efficiency and low contribution to background signal because CDs have intrinsically low Raman activity.

**Fig. 2:**
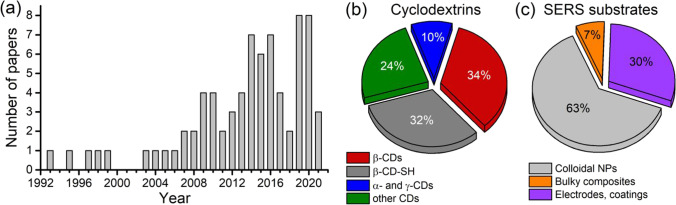
**a** Publication activity in the field of application of CDs in SERS-based studies; 75 papers have been published in total (August 2021). **b**, **c** Distribution of the use of CDs and their derivatives and types of SERS-active parts in CD-SERS analysis

However, despite the growing interest in CD-assisted SERS-based (CD-SERS) systems for the last 10 years (Fig. 2a), there is a lack of summing up information regarding the topic that slows down further progress. Therefore, this report is the first attempt to perform detailed critical analysis of advantages and limitations of the CD-SERS systems published up to date. The review describes (i) CD-containing SERS substrates (CD-SERS substrates); (ii) the use of SERS for physicochemical studies of CD inclusion complexes; (iii) formats of CD-SERS analysis; (iv) the analytical performance of the CD-SERS assays; and (v) advantages, limitations, and perspectives of CD-SERS approaches for the further studies. Therefore, we hope that this review enables to estimate the current state of art of CD-SERS systems and their suitability for the solution of analytical challenges and identify the ways for further improvements.

## CD-SERS substrates

### Cyclodextrins

All primary native CDs (α-, β-, and γ-CD) were tested in CD-SERS analysis (Fig. 2b) and β-CD and its derivatives are the mainly used ones due to the best availability. Thiolated CDs (CD-SH) are the mostly used CD derivatives due to their capability for strong attachment to the metallic surface of SERS substrates. The other CD derivatives studied in CD-SERS analysis are represented, for example, by CD polymers [18], thioether-bridged CD dimer [19], bis-CD diselenide [20], pyridine modified CD [21], CD conjugated with 4-mercaptophenylboronic acid [22], and ethylenediamine-modified β-CD [23].

### SERS-active parts

All types of generally used SERS substrates have been applied for preparation of CD-SERS substrates (Fig. 2c). As in SERS in general, spherical silver and gold nanoparticles (AgNPs and AuNPs, respectively) prepared using wet chemistry approaches are the mainly used substrates due to simplicity and reliability of production. Besides the use for label-free CD-SERS analysis, these substrates were also used for preparation of SERS nanotags [24, 25]. Several reports were dedicated to the fabrication of conductive substrates suitable for electrospectral schemes of analysis, for example, electrochemically roughened metal electrodes [21, 26, 27], vacuum-deposited silver film [28], and silver-modified indium tin oxide electrodes [29]. Quasi one-dimensional anisotropic metal nanoparticles were also tested as SERS substrates [30–32]. Finally, core-shell structures prepared with the use of magnetite particles [33] or polystyrene beads [34] and coated with AgNPs and CD-SH were also tested as CD-SERS substrates. Although copper-based SERS substrates are generally competitive to those based on the noble metals [35], their use for preparation of CD-SERS substrates has not been reported yet. Available information about enhancement factors of CD-SERS substrates demonstrates the common values of the enhancement at 10^5^–10^7^ order. Because the selectivity and extracting capabilities of CD-SERS substrates are of primary interest, the detailed analysis of the enhancement factors is omitted within the paper and the readers are referred to the original reports.

### Preparation of CD-SERS substrates

Modification of SERS substrates by CDs in most of the studies was performed during the post-synthesis step of the fabrication process by CD deposition on a clear surface of the substrate or by substitution of an original stabilizer. One-pot synthesis protocols were also proposed using the mixture of glucose (reducing agent) with native CDs (stabilizer) [36–40]. Finally, the use of pure native CDs for the preparation of SERS substrates by reduction of metal nanoparticles with their further stabilization by excess of CDs has been also proposed [41, 42]. Additional information regarding strategies used for modification of inorganic nanoparticles (including metallic ones) with CD can be found in the review of Gómez-Graña et al. [43].

Summing up the information on the composition and structure of CD-SERS substrates, we can conclude that the progress in material science and preparation of CD-SERS substrates is very high. Thus, we believe that the further studies with CD-SERS systems should be more focused on improvement of understanding of the physicochemical effects responsible for improvement of the sensitivity and selectivity of CD-SERS assays. Also, there are numerous reports dedicated to the preparation of CD-modified metallic nanostructures which were used in the other fields of nanoanalytics (electrochemical, colorimetric, chromatography, etc.), and these reports can also be used as starting points to achieve the best composition for the CD-SERS substrates.

## SERS signal of inclusion complexes with CDs

The first applications of CDs in SERS were aimed to perform physicochemical studies, e.g., improving interaction between target compounds and SERS-active surface or studying the effect of CD complexation on the SERS signal (profile and intensity and their dependence on pH, etc.). Because there are numerous physicochemical results of moderate and minor importance, following, we will discuss only the most important topics which are directly connected with SERS-based analysis.

### SERS signal of native CDs

**Fig. 3:**
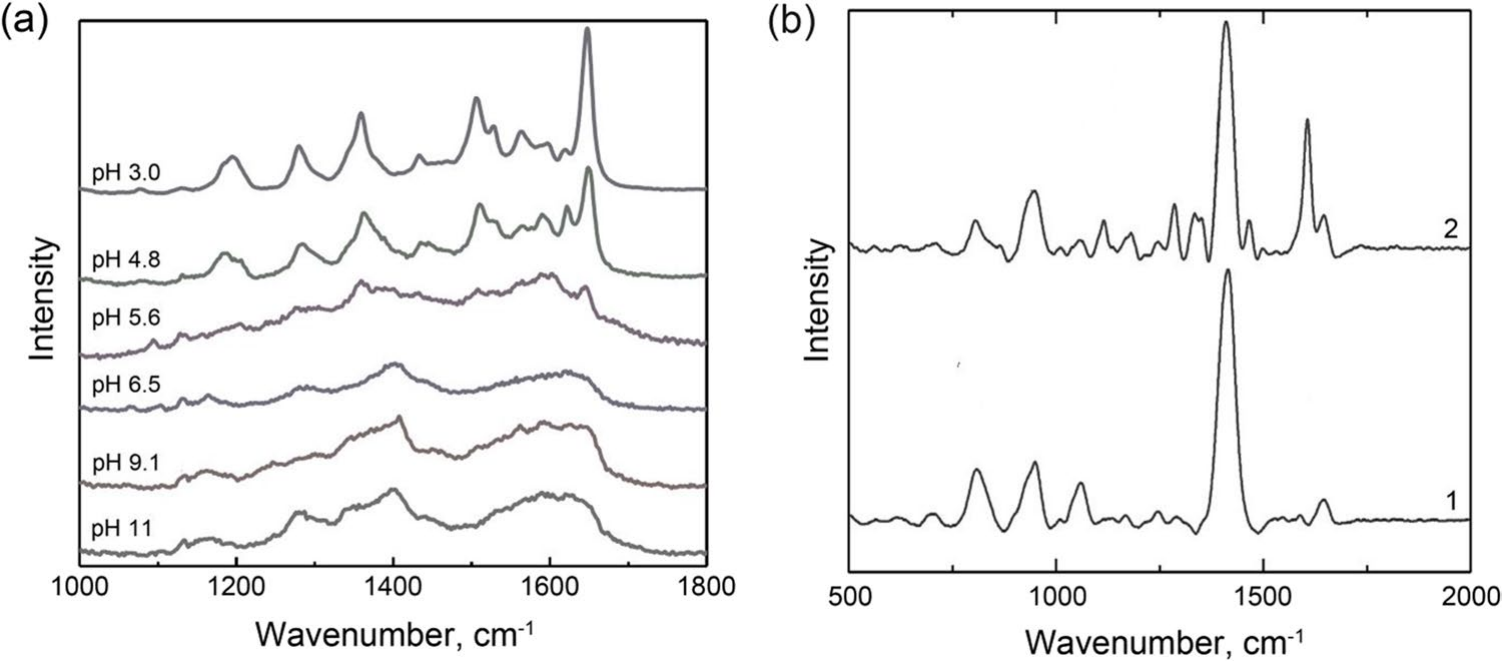
**a** Influence of pH value on the SERS signal of β-CD-modified silver nanoparticles (AgNPs) immobilized inside polyvinyl alcohol matrix. Adapted from ref. [42]. **b** SERS signal of AgNPs stabilized by γ-CD without (1) and with (2) the presence of the analyte (chloramphenicol). Adapted with permission from ref. [41]

Absence of the SERS signal for native CDs is one of the key advantages usually listed in the reports dedicated to CD-SERS analytical systems. However, we found several reports where significant SERS signal of CDs was shown [41, 42, 44]. For example, Ouyang et al. [42] showed that the samples of β-CD-coated AgNPs incorporated in polyvinyl alcohol (PVA) hydrogel have very low SERS signal in neutral and alkaline media but their SERS signal grows significantly in acidic media (Fig. 3a). Unfortunately, the authors did not study the origins of this signal in order to clarify its source (CDs or PVA). Gannimani et al. [41] also found that AgNPs stabilized by γ-CD have intensive SERS signal comparable with that for the analyte (Fig. 3b). The authors explain this signal by the strong binding of the products of CD oxidation to the surface of AgNPs via carboxylic groups formed after oxidation of primary hydroxyl groups of CDs by silver ions. This guess is also supported by the significant negative surface charge of the CD-reduced and CD-stabilized AgNPs (zeta potential is ca. − 30 mW). The negative charge indicates the presence of deprotonated anionic groups on the surface of AgNPs (presumably carboxylic) that is not possible in the case of stabilization by native CDs which do not have such groups. The similar results regarding SERS signal and negative charge of CD-stabilized AgNPs were obtained by de Souza [44].

Remarkably, although CD-AgNPs in three reports [41, 42, 44] were prepared using the same way (reduction of Ag^+^ ions by CDs in alkaline media) and SERS signal was generated using the same excitation wavelength (532 nm), the profiles of their spectra are significantly different (Fig. 3). Moreover, there are numerous papers where a lack of SERS signal of native CDs was stated. Therefore, these discrepancies and the nature of the background signal (and how to control it) have to be thoroughly experimentally investigated in detail in the future because it is particularly important for the development of reliable CD-SERS analytical systems.

Finally, in contrast to native CD, the signal of SERS substrates modified by derivatives of CDs (particularly thiols and amines) has been observed almost always. Despite the obvious negative effect on the analysis reliability, the use of this signal as an internal standard during analysis has been proposed in some reports and this topic is discussed in more detail in the “Ratiometric analysis” section.

### Inhibition of SERS signal in the presence of CDs

Important to note is that there are also several reports where the reduction of SERS signal was caused by CDs [26, 45]. For example, Baretto et al. [26] observed significant reduction of the SERS signal intensity (~ 200 times) after the formation of the inclusion complex. Unfortunately, the authors did not study and analyze this result and the origins of signal reduction are not clear yet. The following effects can be considered to explain the reduction of the SERS signal in the future studies:

(i) The molecules of CDs compete with the analyte molecules (as well as their CD inclusion complex) for adsorption onto the SERS-active sites, reducing the amount of analyte that can interact with the substrate. Consequently, the modification of SERS substrate with CDs should always lead to some reduction of the intensity of the analytical SERS signal compared to the substrate with a clear surface.
(ii) Free analyte molecules and the inclusion complexes have different interaction with the SERS-active surface. For example, the formation of the inclusion complex can occur in bulk solution instead of the SERS substrate surface. The degree and mechanism of adsorption of the inclusion complexes with different “CD–analyte” ratios can also be different leading to the signal reduction.
(iii) CDs can inhibit the Raman enhancement, e.g., by changing the plasmonic coupling between metal particles preventing formation of “hot spots” or changing charge-transfer efficiency between the analyte and metal. For example, the analyte molecules can be capped by more than one CD molecule so that the analyte cannot directly interact with SERS substrate.

Importantly, several mentioned effects can occur simultaneously. For example, Jia et al. [45] also observed a negative effect of CD presence on the SERS signal (up to 2-times decrease of the signal) in the case of excessive CD concentration. The authors used preliminary activation (artificial aggregation) of colloidal CD-SERS substrate to generate “hot spots” and achieve maximal SERS signal. However, Yang et al. [46] showed that the CD can improve colloidal stability against high ionic strength requiring 10 times larger concentration of the aggregation agent. Thus, SERS signal inhibition at increasing CD concentration observed by Jia et al. can be connected not only with blocking of the SERS-active sites by CDs or formation of the inclusion complexes in the bulk solution, but also with too strong stabilization of the metal nanoparticles by CDs and inhibition of their aggregation and formation of “hot spots”.

Considering that CDs have been mainly proposed for enhancement of SERS signal and/or for improving analysis selectivity, these results are very interesting as they demonstrate another side effect of the CD on the SERS signal. Moreover, the decrease of SERS signal caused by presence of CDs can be used to study formation of inclusion complexes [18, 20]. In such case, formation of the complex causes inhibition of the analyte interaction with SERS substrate leading to the signal reduction. For example, Hao et al. [20] used SERS signal to track formation of the inclusion complexes between β-CD and 2,2-diseleno-bis-β-CD (glutathione peroxidase mimicking compound) with some molecules. The authors clearly showed that the formation of the complexes leads to drastic reduction of the SERS signal (4–6 times).

**Fig. 4:**
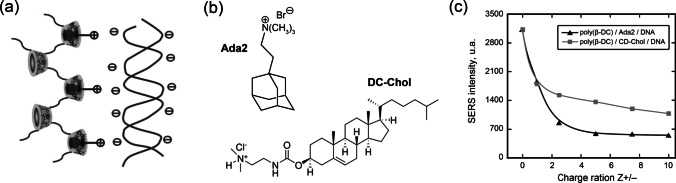
**a** Schematic representation of the complex consisted of DNA molecules attached to poly(β-CD) associated with an amphiphilic cationic connector. **b** Examples of the tested cationic connectors. **c** SERS signal of the adenine residues (1046 cm^–1^) in the complexes with different cationic connectors at various “poly(β-CD)/connectors–DNA” ratios (charge ratios). Adapted with permission from ref. [18]

In the other example, Burckbuchler et al. [18] used the decrease of SERS signal to study conjugation between DNA molecules with poly(β-CD) associated with an amphiphilic cationic connector. Here the SERS signal was used to measure accessibility of the adenyl residues of DNA for interaction with the SERS substrate (AgNPs) after formation of “poly(β-CD)/connector/DNA” complex (Fig. 4a, b). We should note that in this example, CD cavity serves only for capturing the cationic connector and adjusting total charge of the poly(β-CD) and it does not interact with the adenyl residues directly. The experiments with plasmid DNA showed that the increase of “poly(β-CD)/connectors–DNA” ratio (Fig. 4c) leads to decrease of SERS signal intensity. The authors also emphasized that in this study, SERS is the alternative and complementary tool to electrophoretic methods which give information only about the total charge of the complexes. In contrast, SERS provides direct information about accessibility of the free adenine residues of DNA which are not interacting with the “poly(β-CD)/connectors” complex. However, competitive adsorption of the “poly(β-CD)/connectors” on the SERS substrate and its possible contribution to the reduction of the analytical signal due to blocking of SERS-active sites were not considered and estimated by the authors.

### Effect of CD presence in electrospectral systems

Similar to SERS analysis in general, CD-SERS systems were tested for the first time, namely in the electrospectral format [26]. This format implies registration of the SERS signal from the surface of an electrochemically roughened electrode (usually silver or gold) under externally controlled polarization of the electrode. Due to simultaneous registration of the spectral (SERS signal) and electrochemical (*I-V* curves) information, electrospectral systems are an excellent tool to study the effect of CDs on the charge-transfer processes near the SERS-active surfaces and mechanism of the Raman enhancement. However, despite the advantages and high potential, there are only several reports dedicated to electrospectral CD-SERS systems [26, 27] and, unfortunately, none of them is connected with analytical applications.

In the first example, Wang et al. [27] investigated the interaction between native β-CD and electrochemically generated radical intermediate of riboflavin (RF) on the surface of nanoroughened silver electrode. While the effect of CDs on the SERS signal intensity was not analyzed, coupled SERS and electrochemical results show that the presence of CDs shifts reduction potential of RF from − 850 to − 500 mV vs. SCE. The authors concluded that the formation of an inclusion complex between CD and RF can stabilize the anion radical of RF facilitating its further reduction. Accounting that the chemical mechanism of Raman enhancement implies the “metal → molecule” charge-transfer process with formation of short-living anion-radicals [47], these results show usefulness of CDs for investigation of the Raman enhancement mechanism.

**Fig. 5:**
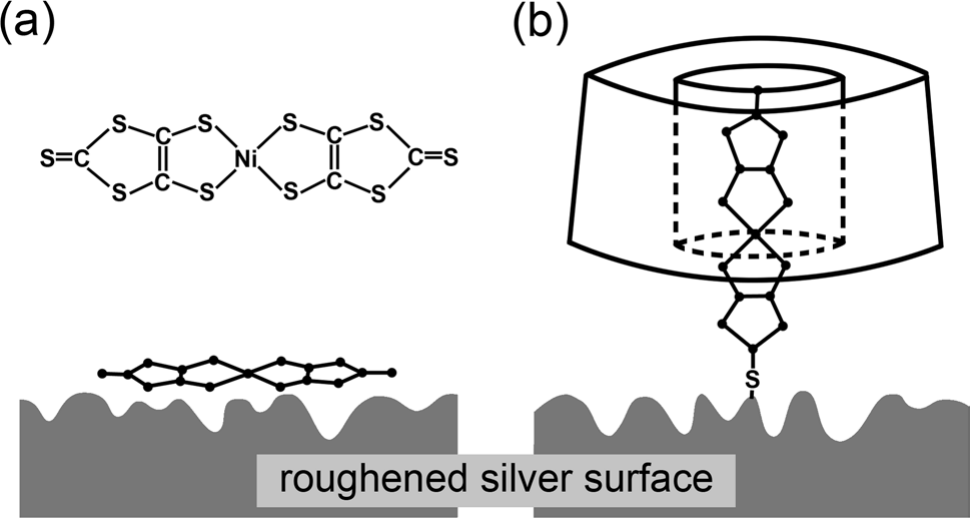
Expected adsorption geometries for **a** neat [Ni(dmit)_2_]^−^ complex and **b** γ-CD-[Ni(dmit)_2_] ^−^ inclusion compound on the rough surface of silver electrode. Adapted with permission from ref. [26]

Baretto et al. [26] used native γ-CD to improve solubility in water of bis(4,5-dimercapto-1,3-dithiole-2-thionato)nickelate(III) anion ([Ni(dmit)_2_] ^−^) and to study the effect of CD on SERS signal of the molecule adsorbed on the silver electrode (Fig. 5). The control experiments without CD were performed using CH_3_CN-H_2_O mixture as a solvent. In the case of the free analyte molecules, the authors observed the increase of SERS signal with moving electrode polarization toward negative values. The opposite was observed for the CD inclusion complex: the shift of the electrode polarization to the negative values leads to decrease of the signal. The authors explained these results by changing orientation of the molecule on the surface of SERS substrate (Fig. 5) that also changes charge-transfer process (i.e., mechanism of Raman enhancement) from “metal → adsorbate” to “adsorbate → metal” during SERS signal generation. However, from our point of view, “adsorbate → metal” charge-transfer model does not seem to be realistic accounting for high electron density in plasmon polaritons generated at nanoroughned metal surface. On the other hand, these results can be explained using only “metal → adsorbate” charge-transfer model [47] accounting that the distance between metal surface and electron accepting part of the molecule (Ni(II) ion) is significantly larger in the case of the inclusion complex compared to free molecule that, consequently, reduces efficiency of electron transfer process.

### Effect of analyte concentration on SERS signal

Because SERS implies adsorption of the analyte molecules onto the SERS-active surface, the dependence of the SERS signal on the analyte concentration can be described as an isotherm of adsorption and this has been successfully shown in several reports dedicated to CD-SERS systems. For example, Yamamoto et al. [48] showed that *o*- and *p*-isomers of methyl red derivative obeyed the adsorption isotherms of the Langmuir and Freundlich types, respectively, during interaction with the CD-SERS substrate. However, Langmuir-type isotherm of adsorption was observed more often [31, 36, 39, 46, 49]. The most reasonable explanation for this fact is homogeneity of the sorption sites due to the use of CDs as the recognition agent with rigid cavity of fixed size. As a consequence, the ranges of linear response of SERS signal on concentration changes are generally quite narrow and include one–two orders of analyte concentrations. On the other hand, the systems with Langmuir-type isotherm of adsorption generally demonstrate better sensitivity (slope of the calibration plot) compared to those with Freundlich type isotherms of adsorption. Also, homogeneity of adsorption sites means reproducibility of calibration plot for CD-SERS assays.

Finishing this section, we should point out that the fundamental, physicochemical studies are very important for the development of new analytical CD-SERS systems and for improvement of the existing ones because they explain behavior and provide control over the systems. Unfortunately, as it can be seen further, fundamental studies of CD-SERS systems fall behind their analytical applications. Therefore, further investigation directed to the understanding effect of the presence of CDs on Raman enhancement mechanism will be very useful. The investigations of the analyte SERS signal inhibition and of the SERS signal of the native CDs are particularly important for maximizing reliability of CD-SERS analytical systems.

## Formats of CD-SERS assays

The simplest format of CD-SERS analysis is the same as in the case of standard SERS analysis—it includes addition of CD-SERS substrate to the analyzed solution with further registration of the analytical signal. Therefore, this section contains description and discussion of only specific, most interesting examples of the assays.

### Ratiometric analysis

In contrast to native CDs, CD-SH generally always have distinct SERS signal due to strong interaction with the metallic surface of the SERS substrates. Although the overlap between the signals of CD-SH and the analyte can complicate analysis and deteriorate its correctness, the CD-SH signal can be used as an internal standard in ratiometric schemes of analysis. Such schemes improve reliability of SERS-based analyte determination due to minimization of negative effect of deviating Raman enhancement for different SERS substrates.

Zhang et al. [50] proposed mesoporous AuNPs modified with β-CD-SH for the determination of polycyclic aromatic hydrocarbons (PAH) (anthracene and naphthalene) and used the signal of β-CD-SH for normalization of the analytical signal. However, no significant improvement of the analysis reliability was found compared to the original signal of the analytes without normalization. Yu et al. [22] also developed a ratiometric CD-SERS assay for PAH determination (anthracene and pyrene). Instead of CD-SH, the authors used a conjugate of native β-CD with 4-mercaptophenylboronic acid (4-MPBA) and the signal of 4-MPBA was used as the internal standard. Importantly, the conjugate was prepared by linking 4-MPBA with CD molecules through OH groups outside the CD cavity that enables to keep the cavity free for the analyte adsorption. Unfortunately, despite the advantages of intensity and clarity, the signal of this internal standard consists of many SERS bands in the range from 400 to 2000 cm^−1^ that limits its applicability due to significant overlap with the analyte signal. Nevertheless, the authors showed that the use of the signal normalization enables to improve accuracy and precision of the analysis. Currently, ratiometric CD-SERS analysis is represented by only these two examples and the further studies are required because none of these reports quantitatively analyzed benefits of the normalization and discussed conditions at which the control signal is stable (pH, ionic strength, etc.).

### CD-SERS analysis with solid-phase extraction

Although SERS always implies solid-phase extraction (SPE) due to requirement for adsorption of analyte molecules on the surface of SERS substrate, we separately describe some examples where SPE step was intentionally used in CD-SERS analysis to concentrate analyte molecules and separate them from the matrix. For example, Vassalini et al. [51] proposed a SERS substrate based on the alginate millimeter-sized particles with embedded AgNPs coated by β-CD. Using methylene blue as a testing analyte, the authors demonstrated that the application of CD-modified AgNPs inside alginate particles enables to improve the SERS signal intensity 15 times compared to alginate particles modified with pure AgNPs. Also, the use of β-CD improves precision of the analysis, reducing RSD from ~ 20 to ~ 10%.

Xu et al. [33] proposed the combination of CD-SERS analysis with magnetic-assisted SPE. The authors developed a SERS-active composite consisted of a magnetic core (Fe_3_O_4_) decorated with the AgNP shell and with the outermost layer of β-CD-SH attached to the silver surface. The composite was used for the determination of butyl benzyl phthalate, an illegal additive in liquors, which does not have suitable functional groups (e.g., amino or carboxyl) in order to get good affinity toward the SERS substrate without the CD coating. The modification of the substrate by CD molecules enables registration of analyte at two-order lower concentrations compared to unmodified SERS substrate. The results show capability of the method to detect the analyte at the concentration levels which satisfy requirements of the normative documentation. Unfortunately, the reliability of the method for real practice is still questionable because the report lacks any details regarding the real object composition and its complexity and lacks verification of the accuracy using control method.

Ouyang et al. [42, 52] developed a CD-containing SERS-active composite consisted of PVA hydrogel with incorporated AgNPs coated with β-CD (PVA-CD-Ag). The composite was used to perform SERS analysis with preliminary SPE step; sibutramine (in pharmaceutical capsules) [42] and sulfonamides (in artificially spiked lake water) [52] were used as testing analytes. Mechanism of the analyte trapping by CD molecules was additionally studied by NMR and fluorescence spectroscopy. Efficiency of the use of CDs was proved showing that PVA-CD-Ag has 50% and 25% better adsorption capability (and, consequently, SERS signal) compared to PVA-Ag toward sulfonamides and sibutramine, respectively. Importantly, besides improved sensitivity, the immobilization of the metal nanoparticles inside porous matrix eliminates possible issues with chemical and colloidal stability in the case of colloidal SERS substrates [53, 54].

Lastly, Wu et al. [32] developed a paper-based SERS substrate which consisted of paper sheets loaded with gold nanorods modified by β-CD-SH. Immobilization of the AuNRs in the porous structure of the paper facilitates the extraction and trapping of the analyte molecules by swabbing the surface of interest with the cyclohexane sodden substrate. The substrate was used to determine hydrophobic dyes Sudan III and Sudan IV which are frequently used as illegal colorants for adulterated foodstuff. Comparison of AuNR-paper with and without CDs shows that the presence of CDs enables analyte detection at 10 times lower concentrations and improves temporal stability of the substrate at the open air conditions over a 3-month period of storage.

### CD-SERS substrates for colorimetric analysis

**Fig. 6:**
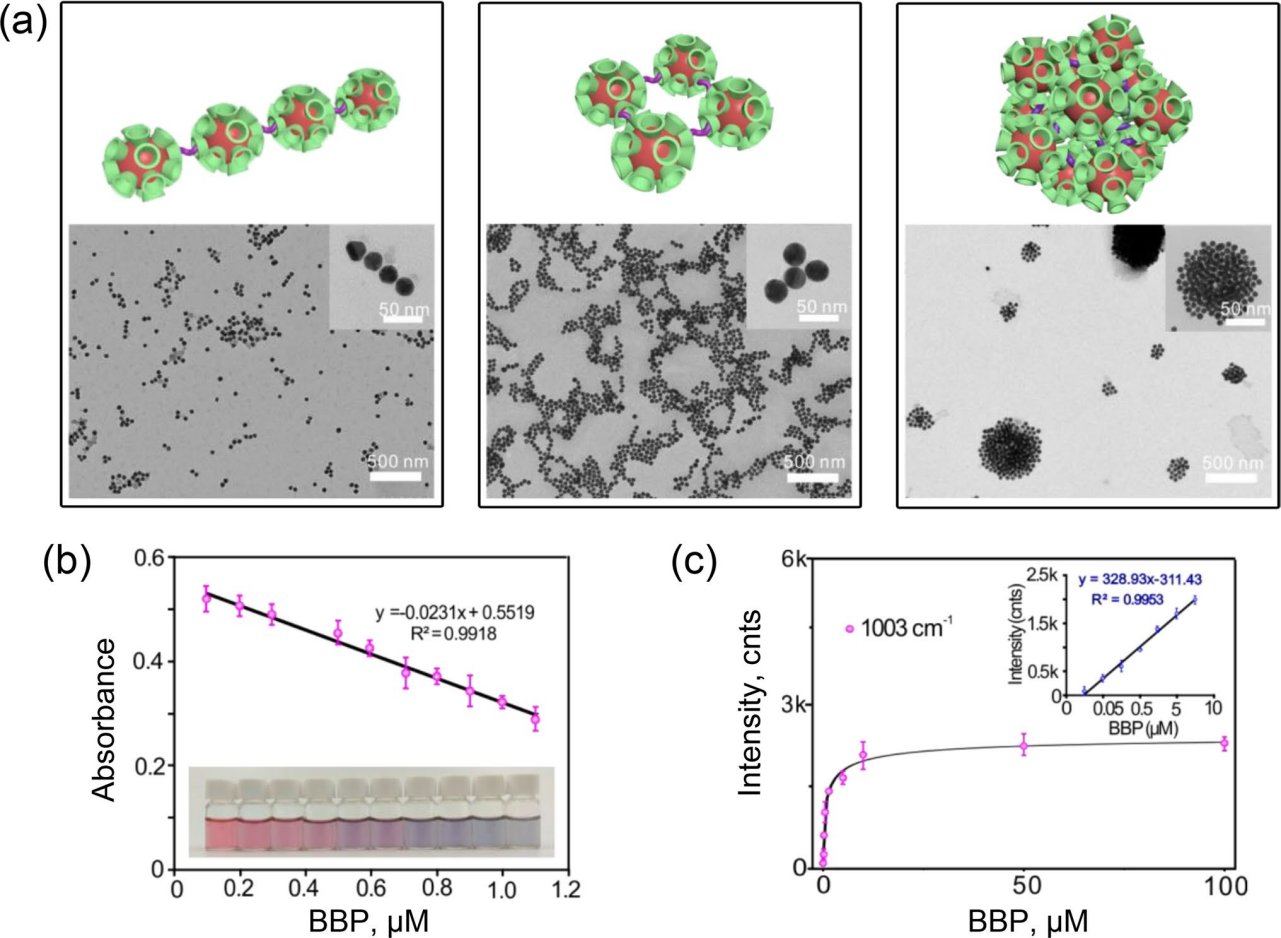
**a** Models of the assemblies of β-CD-AuNPs linked by analyte molecules (butyl benzyl phthalate) and TEM images of the real assembles formed in the analyte solution with different concentrations (0.1, 0.5, and 1.0 μM). Effect of the butyl benzyl phthalate concentration on **b** the absorbance and color and **c** SERS signal of β-CD-AuNPs solution. Adapted with permission from ref. [49]. Copyright 2019 American Chemical Society

Besides the combination of CD-SERS with SPE [33], the group of Xu [49] also proposed an interesting analysis format based on the use of β-CD-AuNPs for both CD-SERS and colorimetric detection schemes (Fig. 6). The structure of the selected analyte (butyl benzyl phthalate) allows for the formation of “CD-analyte” complexes with 2:1 ratio of the components that is favorable for the realization of analyte-triggered agglomeration of the β-CD-AuNPs (Fig. 6a). First, the agglomeration leads to changes of plasmonic properties and, consequently, color of CD-AuNPs and this effect enables analyte determination by measuring changes of absorbance in UV-visible range (Fig. 6b). Second, the agglomeration also favors for increase of SERS signal intensity due to formation of “hot spots” (Fig. 6c). Estimation of the analytical performance shows that both analysis schemes possess adequate figures of merit allowing for the analyte determination in liquor and rice wine. On the other hand, the paper lacks any information about compositions of the real objects and the authors used preliminary liquid-liquid extraction to separate the analyte from the real matrix and concentrate it. Also, the advantages of, namely, dual-mode determination were not highlighted, analyzed, and discussed. However, despite the listed deficiencies and the need for further improvements of the sensitivity and selectivity for both schemes, the combination of the schemes can allow for their cross-verification that can additionally improve reliability of the analysis.

### Reaction monitoring in time and reaction-assisted CD-SERS analysis

**Fig. 7:**
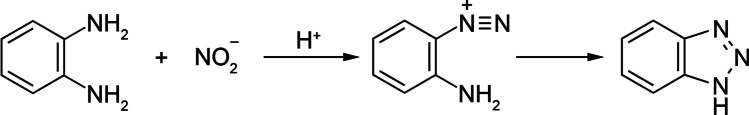
The reaction used to convert o-phenylenediamine (OPD) with nitrite ions to a more SERS-active derivative (benzotriazole)

Generally, SERS signal is collected at equilibrium conditions when any processes connected with the transport of analyte molecules to the SERS-active surface are finished. However, there is no restriction on the use of SERS for dynamic system and Ouyang et al. [55] demonstrated the use of CD-SERS assay to study changes of the analyte concentration within the time course due to reaction process. The authors proposed the use of CD-containing and SERS-active Pickering emulsion as CD-SERS substrate. Paraffin was used as the organic phase of the emulsion, and AgNPs coated with β-CD-SH (CD-S-AgNPs) were used as the stabilizing and capturing agent. The authors investigated two reactions via SERS signal monitoring: (i) formation of benzotriazole from o-phenylenediamine (OPD) and ${\mathrm{NO}}_2^{\hbox{--} }$ (Fig. 7) (both reagents are in aqueous phase) and (ii) oxidation of OPD catalyzed by surface plasmon resonance of CD-S-AgNPs under light illumination (OPD is in the organic phase). In this study β-CD-SH plays two important roles as a multifunctional stabilizer. First, it stabilizes both AgNPs and paraffin droplets allowing for the formation of plasmonic Pickering emulsion and SERS-based monitoring of the interfacial reactions. Second, it serves as a capturing molecule to promote interaction between SERS substrate (AgNPs) and the reaction products via formation of host-guest complex.

Besides the capability to study reaction kinetics, this report demonstrates another interesting approach – triggering of (dis)appearance of the analytical SERS signal as a result of chemical reaction. This format of signal generation in analytical CD-SERS systems was demonstrated by Ma et al. [38] who used α-CD-stabilized AgNPs for determination of OPD. Initially, the authors showed that the analyte has very poor SERS signal forbidding its direct determination. To overcome this issue, the use of a simple chemical reaction to convert the analyte molecules to a more SERS-active derivative (benzotriazole) was proposed (Fig. 7). This derivative demonstrated much better affinity to the CD-SERS substrate enabling its trapping and reliable determination. In the other report, Ma et al. [37] demonstrated reaction triggered disappearance of the analytical SERS signal using reaction between SERS-inactive analyte and SERS-active co-reagent. The authors used AgNPs coated with the «CD – co-reagent» inclusion complex to generate analytical signal. The interaction of the inclusion complex with the analyte molecules leads to formation of a new complex between co-reagent and the analyte which cannot form inclusion complex with CD, presumably due to a too large size. As a result, the growth of analyte concentration leads to decrease of the SERS signal. The authors used this reaction to make a label-based SERS assay with competitive format of analysis and this assay is additionally discussed in the next section. Therefore, the authors of both reports proved the viability of the reaction-assisted CD-SERS analysis that can be very useful for conversion of analytes with poor SERS signal to derivatives with better SERS signal or for indirect analyte determination using the SERS signal of co-reagents.

### Label-based analysis

Although the use of direct (label-free) SERS-based determination of the analytes is dominating, label-based CD-SERS systems have also been reported several times [24, 25, 37, 56]. In this analysis format, CDs are used for the preparation of SERS nanotags that are a SERS substrate with fixed profile of the SERS signal [57]. SERS nanotags are prepared using a Raman reporter—a molecular compound with strong and known Raman signal. Some label-based CD-SERS systems are based on direct interaction between analyte molecules and SERS nanotags via the CD molecules deposited on the surface of SERS substrate [24, 56]. There is also an assay which sensing mechanism is based on the competitive interaction between the analyte molecules and the inclusion complex formed by a CD and the molecules of Raman reporter [37].

**Fig. 8:**
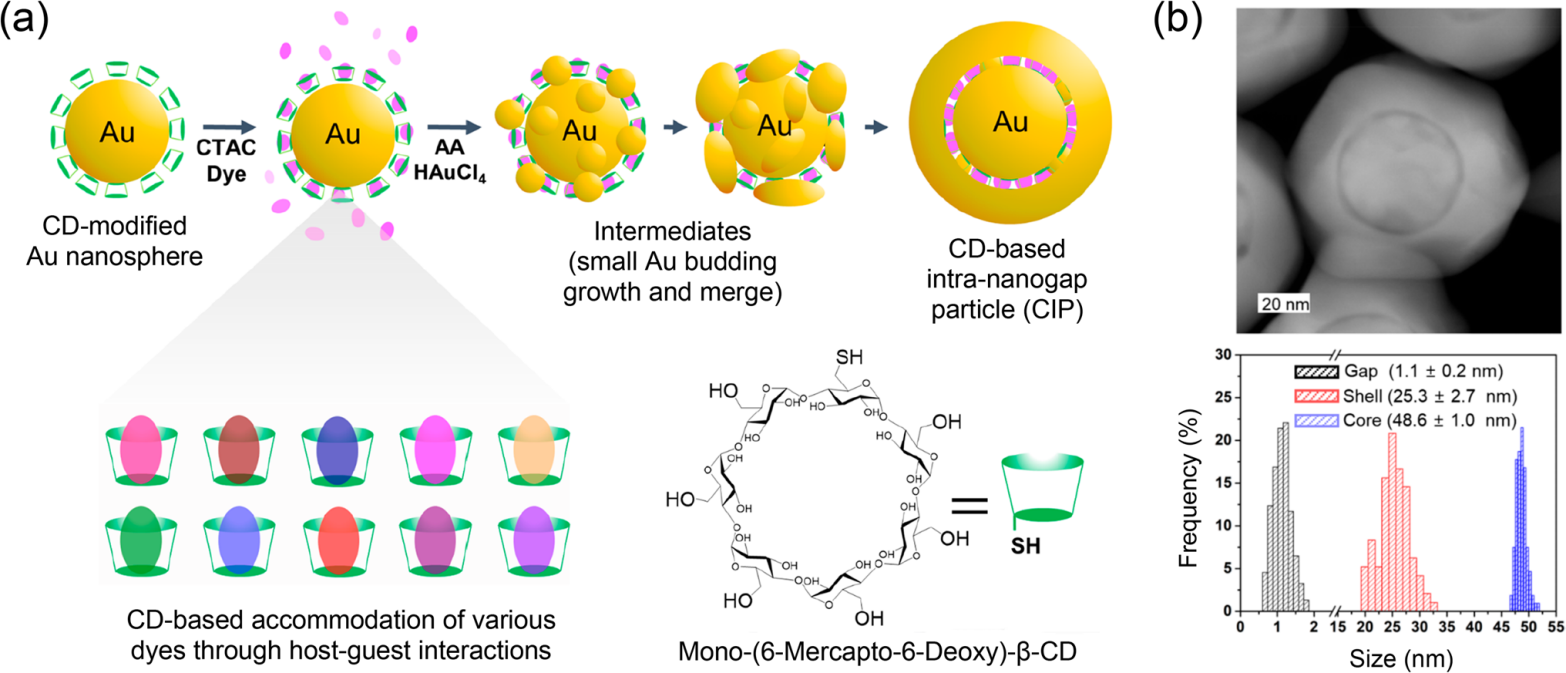
**a** Scheme of the synthesis of cyclodextrin-based intrananogap particles (CIPs) containing various Raman reporter molecules (molecular organic dyes) inside the intrananogap. **b** An example of TEM image of CIP and size distributions of intrananogap, shell, and core inside the CIP. Adapted with permission from ref. [25]. Copyright 2020 American Chemical Society

In the first example, Kim et al. [25] proposed the use of β-CD-SH to maximize loading of the SERS substrate surface by the molecules of Raman reporter (Fig. 8a). Ten different Raman reporters were tested as the reporters to prove the concept of fabrication scheme. Additionally, the authors deposited a metallic shell over the CD layer loaded by the reporter molecules that enables achievement of the maximal SERS signal due to formation of numerous “hot spots.” The resulting CD-based intrananogap particles (CIPs) with the metallic core-shell structure (Fig. 8b) were additionally modified with antibodies and used as SERS nanotags in the multiplex SERS-based cell imaging. Importantly, in this example, β-CD-SH is not involved in the analysis step directly and it is used only to improve the overall signal of SERS nanotags due to (i) formation of distinct gap between the core and the shell and (ii) maximization of the nanotag loading by the Raman reporter via permanent formation of the inclusion complexes. We should note that besides improving signal intensity, core-shell architecture also enables to avoid competitive interactions on the surface of SERS substrate between the “CD-Raman reporter” complex and the components of the analyzed object. Unfortunately, no reliable comparison of Raman enhancement efficiency of CIPs with the SERS nanotags of other structures was done, e.g., comparison with single-core nanotags or the nanotag-based di- and trimeric aggregates.

**Fig. 9:**
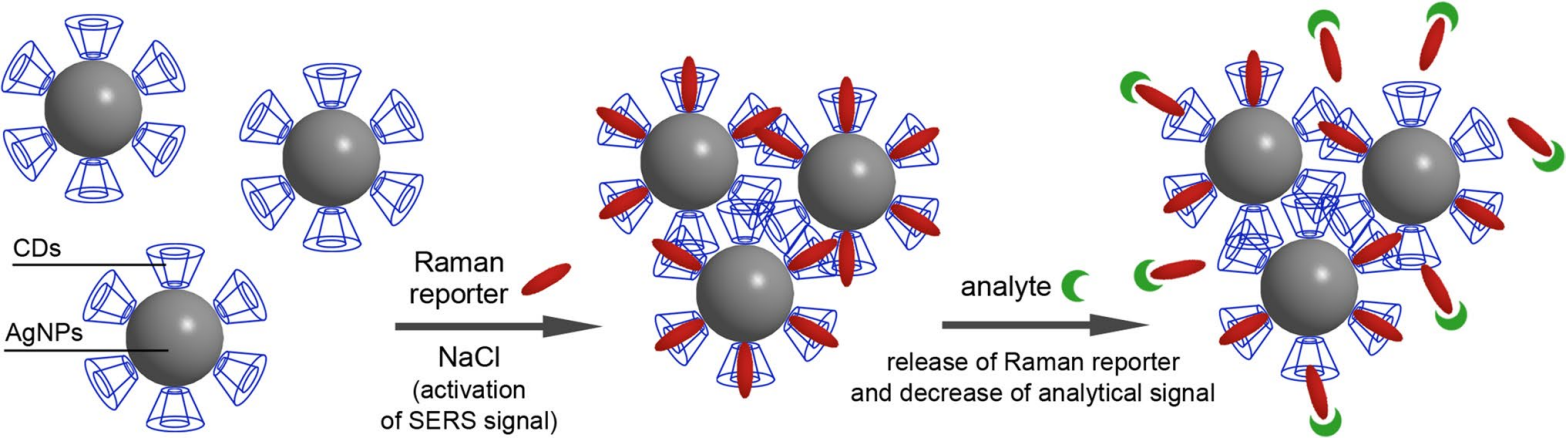
Schematic representation of competitive format analysis using SERS nanotags based on silver nanoparticles coated with “CD-Raman reporter” complex

In the next example, Ma et al. [37] realized a competitive format of analysis for indirect determination of mercury ions using AgNPs coated with the “CD-Raman reporter” inclusion complex. The mercury ions break down the inclusion complex due to stronger interaction between the ions and the Raman reporter molecules (methimazole) leading to reduction of the analytical signal (Fig. 9). Besides achieving figures of merit satisfactory for practical applications, the authors also demonstrated appropriate selectivity toward 11 other metal ions.

**Fig. 10:**
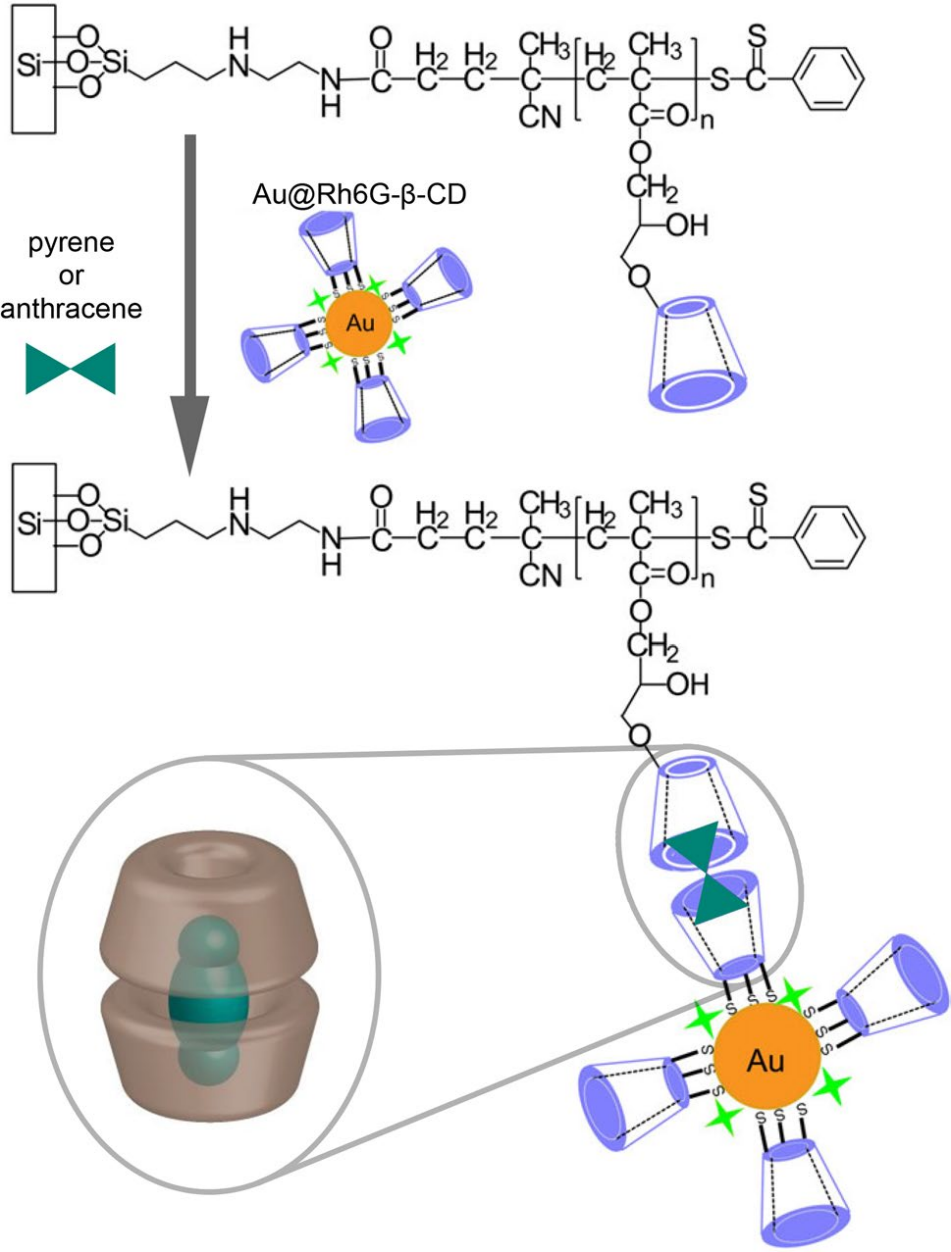
Sandwich-type assay for CD-SERS-based determination of pyrene and anthracene; gold nanoparticles coated with rhodamine 6G and β-CD-SH (Au@Rh6G-β-CD) were used as a label for signal generation. Adapted with permission from ref. [24]

Zengin et al. [24] proposed a sandwich-type assay for the determination of pyrene and anthracene using SERS nanotags and polymeric compound both of which are modified with CDs (Fig. 10). At the first step, the analyte was extracted from the solution using silicon substrate coated with the β-CD-modified poly(glycidyl methacrylate) brushes. Then, SERS nanotags based on AuNPs functionalized with β-CD-SH and Raman reporter (rhodamine 6G) were assembled with the extracted analyte molecules onto the surface of the silicon substrate with the following registration of the analytical signal. The authors also showed that the assay has appropriate selectivity toward some other interferences (coronene, triphenylene, toluene, xylene, naphthalene, and phenylenediamine).

Finally, Wang et al. [56] developed a label-based stereoselective CD-SERS assay and used it for the recognition of tryptophan, phenylalanine, and tyrosine enantiomers. The used CD-SERS substrate consists of AgNPs deposited over TiO_2_ nanoparticles and coated with β-CD-SH and *p*-aminothiophenol (*p*-ATP). β-CD-SH was used as a primary recognition molecule for the discrimination of *S*- and *R*-enantiomers of the analytes, while p-ATP was used to improve the recognition and to generate the analytical signal (i.e., it is a Raman reporter). The use of the TiO_2_ nanoparticles was proposed to improve efficiency of the charge-transfer processes between AgNPs, p-ATP, and the “analyte-CD” inclusion complexes during excitation of surface plasmon resonance in AgNPs. Consequently, this improvement influences the charge-transfer mechanism of Raman enhancement. Importantly, the authors tested several excitation wavelengths (532, 633, and 785 nm) and showed that proper choice of the excitation energy plays a key role for efficient stereoselectivity. The use of photons with high energy (532 and 633 nm) results in the similar efficiency of Raman enhancement for both enantiomers, while the remarkable difference in the enhancement was observed using the low energy excitation. The authors explain this result by the decrease of charge-transfer efficiency in the case of one of the enantiomers due to the formation of weaker hydrogen bonds between the analyte molecules and p-ATP. Therefore, because the difference in the hydrogen bond energy for both enantiomers is small, the difference in the Raman enhancement can be observed only in the case of low energy excitations. Additionally, this participation of hydrogen bonds between p-ATP and the analyte explains the role of p-ATP in the improvement of the analyte recognition (i.e., stereoselectivity). However, while showing promising results, further evaluations of the assay in the analysis of mixtures *S*- and *R*-isomers and admixtures are required as well as capability to discriminate between *S*-isomers of different molecules.

### Gas-phase analysis

A single report on the use of CD-SERS analysis for the determination of gaseous compounds was published by Hill et al. [28]. The authors developed planar CD-SERS substrate based on nanoroughened silver film coated with heptakis(6-thio-6-deoxy)-β-CD and used it for the detection of a series of aromatic compounds (benzene, toluene, ethylbenzene, m-xylene, chlorobenzene, m-dichlorobenzene, anthracene, naphthalene). While an unmodified SERS substrate enables signal registration only at very high analyte concentrations (saturated vapors), the CD-SERS substrate enables signal registration down to tens of ppm. The authors also showed that CD-SERS substrate has its own signal because of use of the thiolated CD and that the analytical signal is the superposition of the signals from CD modifier and adsorbed analyte molecules.

The key feature of this system is the reversibility of signal response potentially enabling continuous monitoring of the concentration changes. However, most of the analytes (except for benzene) showed very slow desorption that limits the capability of the sensor for real-time concentration monitoring. Thus, the authors proposed it for accumulative detection mode or to track slow changes of the concentrations (hours). Interestingly, the authors also found that the sensing capability can be recovered relatively fast by rinsing of the substrate with adsorbed analyte by pure water and subsequent drying, and the covalent binding of CD to the SERS substrate is particularly useful in this case.

Concluding the section, we should state satisfactory progress in the implementation of diverse formats in the CD-SERS analysis and generally better development of analytical applications of CD-SERS systems compared to fundamental studies. However, the number of good analytical studies is still relatively small and additional investigations will be helpful for realistic estimation of pros and cons of CD-SERS assays.

## Analytical performance

### Analytes and figures of merit

Examples of the analyte molecules determined using CD-SERS assays and the information about the assays and their performance are shown in Table 1. First of all, we should note that, unfortunately, the standard figures of merit (limit of detection (LOD), relative standard deviation (RSD), and apparent recovery) have been rarely used for characterization of the analytical performance of CD-SERS assays.

**Table 1:** Examples of the CD-SERS systems proposed for the use in chemical analysis. Abbreviations used in the table: *RSD* relative standard deviation, *PVA* polyvinyl alcohol, *ITO* indium tin oxide

Analyte	CD	SERS substrate	SERS measurement conditions	Analyte concentrations (μM)	LOD (μM)	RSD (%)	Ref.
*Drugs*							
Marbofloxacin	β-CD	AgNPs@CD	785 nm, 150 mW, 10 s	0.003–0.03	0.0017	–	[40]
Sulfamonomethoxine Sulfadiazine Sulfadimidine	β-CD	PVA hydrogel doped with AgNPs@CD	785 nm, 100 mW, 3 s	0.36–36 0.4–40 0.36–36	–	< 10	[52]
6-Mercaptopurine	β-CD	AgNPs@CD	785 nm, 105 mW, 10 s	0.004–0.2	0.0024	–	[46]
Sibutramine hydrochloride	β-CD	PVA hydrogel doped with AgNPs@CD	532 nm, 3 mW, 2 s	22–474	9.5	< 10	[42]
Luteolin	Ethylenediamine-modified β-CD	SiO_2_@Ag nanoparticles modified with CD	532 nm, 5 mW, 2 s 10× objective	0.1–1000	–	–	[23]
*Pollutants*							
Butyl benzyl phthalate	β-CD	AuNPs@CD	633 nm, 0.9 mW, 5s	0.01–10	–	–	[49]
o-Phenylenediamine	α-CD	AgNPs@CD	785 nm, 150 mW, 10 s	0.1–1	0.03	–	[38]
Polychlorinated biphenyls	β-CD-SH	ITO electrode coated with Ag nanostructure	532 nm	0.3–30	–	< 15	[29]
Anthracene naphthalene	β-CD-SH	AuNPs@CD	633 nm, 2 mW, 10 s 100× objective	0.0006–5.6 0.0046–46	– –	7–10 –	[50]
Pyrene anthracene	β-CD conjugated to 4-mercaptophenylboronic acid	AuNPs@CDs	633 nm, 2.3 mW, 10 s 20× objective	0.002–0.01 0.01–0.1	0.0004 0.0044	– –	[22]
Perylene	thioether-bridged β-CD dimer	SiO_2_@Ag nanoparticles modified with CD	532 nm, 10 mW, 5 s 10× objective	0.1–10000	–	–	[19]
Malachite green	β-CD	AgNPs@CD	785 nm, 150 mW, 10 s	0.027–0.41	0.0027	–	[45]
Mercury ions	α-CD	Methimazole-functionalized AgNPs@CDs	785 nm, 150 mW, 1 s	0.001–0.75	0.0005	–	[37]
*Pesticides*							
Paraquat diquat difenzoquat	β-CD-SH	CD-decorated gold microparticles	785 nm, 5 mW, 5 s	0.27–537 0.14–291 0.2–402	– – –	4–8	[61]
Carbendazim	β-CD-SH	AuNRs@CD	785 nm, 0.8 mW, 15 s 50× objective	20–200	50	–	[30]
Methyl parathion	mono-6-thio-β-CD	AuNRs@CD	632.8 nm, 25 mW, 12 s	10^-6^–1	–	–	[31]
*Illegal additives*							
Sudan III Sudan IV	β-CD-SH	Filter paper coated with AuNPs@CD	632.8 nm, 1 mW, 5 s 50× objective	0.1–100 0.5–50	– –	– –	[32]
Melamine	α-CD	AgNPs@CD	785 nm, 150 mW, 10 s	0.04–0.79	0.02	–	[36]
Phenformin hydrochloride	β-CD	AgNPs@CD	532 nm, 60 s	0.07–1	0.008	–	[39]

The examples in Table 1 also show that the mainly used analytes for CD-SERS determination are the aromatic compounds or the compounds with aromatic moieties. Besides high affinity to the CD cavity due to low polarity and well fitting with the CD cavity size, such analytes also have intrinsically high Raman activity (compared to aliphatic compounds) due to conjugated π-electron systems and large polarizability. Namely, this fact enables to get high SERS signal and achieve the best LOD values. Therefore, the lack of reports dedicated to direct CD-SERS determination of aliphatic analytes can be explained by inappropriately low intensity of the analytical signal that starts to be comparable with the level of background signal formed by solvent, admixtures, and even capturing agent (i.e., CD-SH molecules). Also, molecular dyes were widely used as testing analytes for physicochemical studies and preliminary testing of CD-SERS substrates, namely, due to high Raman activity [26–28, 48, 58]. For example, SERS was used to study the interaction of methyl orange with CDs and to investigate the effect of the inclusion complex formation on protonation and stability of azo form of the dye [48, 58]. The dyes were also used as Raman reporters in some label-based CD-SERS assays [24, 25]. However, despite the availability of several relevant reports [32, 45], the determination of dyes in the real samples is of minor interest and it was not analyzed in this review.

### Enhancement of SERS signal in the presence of CDs

Achieving the maximal values of the analytical signal and the signal-to-noise ratio is one of the main tasks during development of any analytical system including CD-SERS assays. Therefore, the use of CDs was also proposed to increase the analytical signal by improving the interaction between analytes and SERS substrates. Unfortunately, most of CD-SERS reports do not contain comparative studies with the control substrates in order to prove superiority of the CD-SERS assays compared to the assays without CDs and we will discuss the exceptional reports in this part.

The improvement of the SERS signal intensity due to CDs was qualitatively demonstrated by Ouyang et al. [42, 52]. The authors showed that the SERS signal of the analytes (sibutramine [42] and sulfonamides [52]) appears only after modification of AgNPs with CD molecules (discussed in the “CD-SERS analysis with solid-phase extraction” section). Quantitative estimation of CD effect on SERS signal in comparison with citrate-stabilized AgNPs showed that CD-AgNPs can provide around 3 times larger signal of melamine [36] and 4 to 8 times larger signal of 6-mercaptopurine (anticancer drug) [46]. On the other hand, Yang et al. [46] also showed that CD modification of AgNPs led to two times reduction of the linear range of concentrations for 6-mercaptopurine. However, 4 to 8 growth of the SERS intensity and 2-fold increase of the sensitivity coefficient (i.e., slope of the calibration plot) resulted in the improvement of sensitivity and accuracy of the determination that is particularly critical for the determination of drugs with narrow therapeutic window (like anticancer drugs).

Remarkable enhancement of SERS intensity and extending the range of detected concentrations were also successfully demonstrated for CD-SERS substrates compared to pure metallic surface [19, 33]. For example, Zhou et al. [33] showed that the use of AgNPs deposited over Fe_3_O_4_ particles and modified by CD-SH enables to extend analytical range of concentrations for two orders of magnitude (from 10^–6^ to 10^–8^ M) compared to the substrate without CDs. The same effect of CD modification on the analytical range was observed by Hahm et al. [19] during the determination of polycyclic aromatic hydrocarbons using CD-AgNPs deposited over SiO_2_ nanoparticles.

However, the limited number of relevant studies does not allow to state that CD-based signal enhancement is a mandatory effect. Indeed, in the “Inhibition of SERS signal in the presence of CDs” section, we have already discussed some cases where the presence of CDs can even cause deterioration of the SERS signal intensity. Also, false signal enhancement can be observed if the CD molecules on the SERS substrate surface are replaced by the analyte molecules easier compared to stabilizer molecules of the control SERS substrate (e.g., citrate ion). Therefore, the formation of the “CD-analyte” inclusion complex and its adsorption on the SERS-active surface should be carefully and critically studied in any particular case.

### Selectivity

Together with improvement of the signal intensity, the use of CDs may improve selectivity of SERS analysis due to (i) limited size and strict shape of the CD cavity and (ii) low polarity of the cavity. Size-exclusion effects lead to inability for too large and too small molecules being efficiently captured by CD-SERS substrates. In contrast to charged stabilizers (e.g., citrate or borohydrate), poor capturing is also expected for polar analytes which interact with a solvent (usually water) much better than that with the CD cavity. The same selection rules are also relevant for the molecules of potential interferences and this fact should be taken into account during evaluation of the selectivity. However, the choice of the interfering compounds and their concentrations was not adequately justified in most of the reports leading to the questionable reliability of the proposed CD-SERS assays for real sample analysis.

Remarkably, the effect of metal ions has been investigated most often [37, 39, 40, 46]. However, from our point of view, such interferences are not quite reasonable (particularly at low concentrations) because they have poor interaction with the CD cavity due to different polarity and do not have Raman signal. On the other hand, there are some exceptions which can justify the need for accounting the presence of the metal ions. First, high ionic strength of the analyte solution can influence solubility of the analyte molecules and their interaction with the CD cavity. Second, the evaluation of the ion effect is mandatory when an interaction between the ions and the analyte molecules is significant and can lead to breaking down “CD-analyte” complexes. For example, Ma et al. [37] used strong interaction between the Hg(II) ions with the molecules of Raman reporter to generate analytical signal (discussed in detail in the “Label-based analysis” section). Third, because matrices with intrinsically high ionic strength (marine water, urine, etc.) significantly influence colloidal stability of the colloidal SERS substrates, such influence has to be accounted and investigated in advance to achieve maximal reliability of the final analysis protocols. Remarkably, Yang et al. [46] showed that the CD-coated AgNPs require one order larger concentration of the activation agent (NaCl) compared to the citrate-stabilized AgNPs in order to trigger the aggregation process. This result shows that the use of CDs can also be beneficial for improvement of stability of the colloidal SERS substrates. In all other cases, the need for investigation of the ion effect is quite questionable.

CD-SERS assays were tested for the analysis of several complex objects: pharmaceutical forms [39, 42, 46, 59, 60], milk and milk powder [36], apple extract [61], soil samples [22], environmental water [37, 38, 45, 52], artificial human urine [60], and alcoholic drinks [33, 49]. The figures of merit for some of these systems are shown in Table 2. However, the effect of interferences and co-existing components (most relevant for the object of interest) was investigated in detail only in few reports [36, 42, 60]. Verification of the CD-SERS assays using control methods was also rarely employed [22, 42, 59]. Moreover, some reports suffer from faulty methodology, e.g., performing the artificial spiking of the samples after significant sample pretreatment (extraction and dilution of the extract).

**Table 2:** Examples of the application of the CD-SERS assays for the analyte determination in real matrices. More information and the other figures of merit for these examples are provided in Table 1

Matrix	Analyte	Number of samples	Recovery (%)	RSD (%)	Ref.
Pharmaceutical forms	6-Mercaptopurine	2	102–103	< 4	[46]
	Sibutramine hydrochloride	6	90–110	–	[42]
	Phenformin hydrochloride	4	95–105	< 6	[39]
Environmental water	o-Phenylenediamine	4	98–104	< 4	[38]
	Sulfamonomethoxine	1	93	–	[52]
	Sulfadiazine	1	114	–	
	Sulfadimidine	1	94	–	
	Malachite green	1	85–103	< 6	[45]
	Mercury ions	4	98–105	< 4	[37]
Alcoholic drink	Butyl benzyl phthalate	2	87−109	< 8	[49]
Soil samples	Pyrene	2	102–102	< 2	[22]
	Anthracene	2	102–106	< 3	
Milk powder	Melamine	4	–	< 7	[36]
Milk		3	89–104	< 5	

Despite the growing interest in the application of SERS for analysis of body fluids, the use of CD-SERS assay in this direction was proposed only by Cao et al. [60]. The authors used commercial SERS substrate modified with β-CD-SH for the determination of acetyl amantadine (a cancer biomarker) in urine. Unfortunately, artificial urine samples were used in the study, but the assessment of the selectivity was performed quite critically trying to estimate real capabilities of the CD-SERS assay. For example, the authors demonstrated that the modification with β-CD-SH does not enable elimination of the negative effect of some metabolites (creatinine and corticosterone) which remarkably compete with the target analyte reducing its SERS signal. However, because the analysis of body fluids by SERS is really a challenging task due to strong competitive interactions with intrinsic body fluid components [5, 7], more studies in this direction (particularly with real samples) are required before concluding the suitability of CD-SERS assays for such analysis and capability of CDs to improve selectivity.

The selectivity of CD-SERS substrates to the compounds with closely related structure to the target analyte was also investigated in several reports. For example, high selectivity of the CD-SERS substrates was demonstrated for methyl parathion insecticide vs. some other organic pollutants (mirex; phthalocyanine; hydroquinol; resorcinol; and 1,3-phenylenediamine) [31], OPD vs. several other arylamines [38], luteolin vs. other flavonoids (hesperetin, naringenin, quercetin) [23], and pyrene and anthracene vs. other (poly)aromatic compounds [19, 24]. On the other hand, Ouyang et al. [52] demonstrated that their CD-SERS substrate has equal affinity to several sulfanilamide antibiotics (sulfamonomethoxine, sulfadiazine, sulfadimidine). This fact can be connected with the realization of the similar mechanism of the “CD-analyte” complex formation via sulfanilamide group. In this case, poor selectivity to the compounds with similar structures can be considered an advantage which can be used for development of the protocols suitable for class-specific analysis.

Finally, we should note that there is still a lack of studies demonstrating that CDs can improve selectivity compared to control SERS substrate with blank (pure) metallic surface or with often used stabilizers (e.g., citrate ions). Also, accounting that the presence of CDs can reduce SERS signal under some circumstances (discussed in the “Inhibition of SERS signal in the presence of CDs” section), we believe that CDs can also be tested for masking of admixtures and suppressing their SERS signal. In this case, the interaction of CDs with the admixtures should be stronger compared to that with the target analyte molecules (e.g., due to size and polarity differences). On the other hand, the interaction between the “admixture-CD” complex and the SERS substrate surface has to be weaker compared to the interaction between the substrate and pure analyte molecules. If these requirements are achieved, the presence of CDs can help to suppress background signal improving accuracy and precision of the analyte determination. Such effects can be particularly useful for analysis of complex mixtures with strong background signal such as body fluids and food products.

### Stereoselectivity

As mentioned in the “Introduction” section, CDs are widely used in chemical analysis for separation and recognition of isomers of optically active compounds (enantiomers) [15–17]. Thus, stereoselectivity of the CD-SERS systems has also been investigated in several reports [34, 48, 59, 62]. Discrimination of the enantiomers of some amino acids was performed by Wang et al. [56] using label-based format of CD-SERS analysis, and this report has been already discussed in detail in the “Label-based analysis” section; all other studies were performed using label-free format of CD-SERS analysis and they are discussed here.

Yamamoto et al. [48] used colloidal AgNPs modified by 6-(2-mercapto-ethylamino)-6-deoxy-α-CD (MEA-α-CD) to study SERS signal of p- and o-methyl red isomers conjugated with enantiomers of 1-phenyl-ethylamine (o-MR-PEA and p-MR-PEA, respectively). First of all, the authors showed that MEA-α-CD and native α-CD have similar stereoselectivity demonstrating better interaction with *R*-enantiomers of o-MR-PEA than with the *S*-enantiomer. Importantly, the interaction between the analytes and MEA-α-CD was found stronger that additionally improves sensitivity of the CD-SERS analysis. Formation of the more stable complex between o-MR-PEA and MEA-α-CD shows that this CD-SERS system is also regioselective. However, the authors did not discuss changes of the spectral profiles in the case of different enantiomers that are critical for discrimination of the enantiomers using SERS. Also, they did not investigate the mixtures of the isomers (stereo- or regio-), so the suitability of this CD-SERS system for the analysis of the mixtures has not been proved.

Abalde-Cela et al. [34] used polystyrene beads coated with AgNPs and β-CD-SH for chiral recognition of *R,R*- and *S,S*-hydrobenzoin. The analyte has very poor affinity to coinage metal surfaces that complicates direct registration of its SERS signal and this fact was consciously used by the authors to demonstrate capability for molecular trapping by the CD layer. While the positions of SERS bands for the different enantiomers were the same, the spectral profiles were significantly different and SERS intensity in the case of S,S-enantiomer was better (up to 20%). Finally, the authors successfully demonstrated applicability of their CD-SERS assay for the recognition of the enantiomers and semiquantitative analysis of racemic mixtures. The only disadvantage of this study is the use of quite high concentration of the analytes (1 mg mL^−1^).

**Fig. 11:**
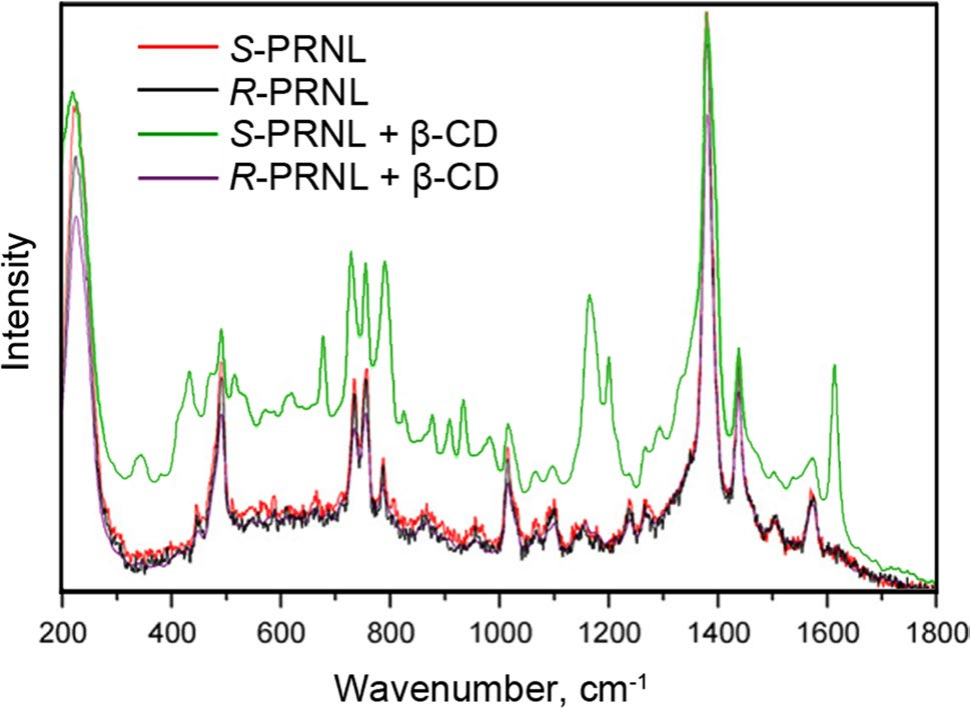
SERS spectra of propranolol enantiomers (*R*- and *S*-PRNL) in the absence and presence of β-CD. Adapted with permission from ref. [59]

Bodoki et al. [59] proposed a CD-SERS assay based on colloidal AgNPs coated with native β-CD for the discrimination of the propranolol enantiomers. The authors demonstrated significant changes of the SERS signal in the case of *R*-propranolol (Fig. 11) and used multivariate regression analysis to treat results. The error of prediction was found below 1.6% and the results of CD-SERS analysis of the enantiomer mixture in pharmaceutical formulations (tablets) were verified by chiral HPLC. In the other reports [62], the authors additionally investigated recognition of the propranolol enantiomers by CD-SERS systems and found that β-CD showed the best results compared to α- and γ-CDs. Importantly, incubation of the reagent mixture for at least 60 min is required before the signal registration to achieve the maximal signal and to reach the equilibrium. Lastly, the importance of the reagent addition sequence was also demonstrated and requirement for preliminary formation of the “CD-analyte” complex before addition of the SERS substrate was shown.

Therefore, these examples show that CDs and their derivatives remain stereoselective after immobilization onto the surface of SERS substrates making CD-SERS assays suitable for chiral recognition.

## Advantages and limitations

### Advantages

First, CDs are cost-effective (particularly β-CD), stable, and have known and constant cavity size that positively influences reproducibility of the results. Second, CDs enable to improve solubility of hydrophobic compounds in water due to the formation of the inclusion complexes. This advantage is particularly important if to account that SERS is a surface-related spectroscopy and requires transport and binding of the analyte molecules to the SERS substrate surface. Third, for the SERS substrates based on colloidal metal nanoparticles, the use of CDs can improve colloidal stability of the substrates in samples with high ionic strength [46]. Finally, the most important advantage of CDs is their capability to modulate interaction between the SERS substrate and the molecules of interest enabling improvement or deterioration of the interaction between them (and, consequently, intensity of SERS signal) [18, 22, 49, 50, 59]. This capability is useful in the case of molecules without functional groups responsible for the strong binding to the metallic surface of the SERS substrates [22, 49, 50]. Additionally, CDs can influence the orientation and protonation of the analyte molecules on the surface of SERS substrate that also influences Raman enhancement [26, 48, 58]. Namely, the possibility to control the “analyte-substrate” interaction together with quite low strength of the “analyte-CD” interaction (compared to «antibody-antigen» interaction) enables realization of diverse analytical formats with CD-SERS substrates making CD-SERS analysis a flexible analytical tool.

### Limitations

Because of the general tendency to overestimate advantages and underestimate limitations, this part of the review is the most difficult one. However, adequate estimation of the limitations is critical for developing reliable analysis protocols and for distinguishing the most interesting directions for further work. The primary limitation of CDs is the worse selectivity compared to biological recognition elements such as antibodies and aptamers. Indeed, according to the examples shown in Table 1, the same CD has been proposed to improve determination of diverse analytes. Fortunately, the multiband nature of the SERS signal partially compensates this limitation reducing the chance of false-positive results by distinguishing the signal of analytes and unexpected admixtures using analysis of the spectral profiles (particularly using multivariate data analysis). Moreover, the restricted selectivity allows for the development of class-specific protocols using a single recognition molecule and analysis protocol [22, 52].

Importantly, the CD selectivity is also restricted by size-exclusion effects due to rigid structure and limited size of the CD cavity. Therefore, the analyte molecules with too large size or without suitable functional groups (which can fit cavity size) cannot be trapped by CDs because of inability to form inclusion complexes. On the other hand, size-exclusion effects can be tested for suppression of background SERS signal, e.g., by separation of small analyte molecules from admixture molecules with large size.

Besides selectivity, the interaction between CDs and analyte molecules are also weaker compared to that in “antigen-antibody” complexes leading to the reduction of the analysis sensitivity. However, high sensitivity of SERS technique can partially compensate for deterioration of the sensitivity of CD-SERS assays. Also, in some cases, the interaction between CD and analytes is significantly better compared to that for the pure metallic surface of SERS substrate (particularly for analytes without polar functional groups). Finally, reversibility of the inclusion complex formation can facilitate the development of SERS sensors enabling continuous monitoring of the analyte concentration changes in time.

The last and rarely accounted limitation is the possible blocking of SERS-active sites on the substrate surface by deposited CD molecules that can cause the deterioration of Raman signal enhancement. Reduction of SERS signal can also appear in the case of high excess of CDs and formation of the inclusion complexes in the bulk solution instead of the SERS substrate surface [18, 20, 26, 45]. Finally, CDs can be a source of background signal, particularly in the case of thiolated CD derivatives [22, 41, 42, 44, 50]. However, the SERS signal of CDs was proposed for the use as an internal standard in ratiometric assays [22, 50], but additional experimental studies are required to prove efficiency and viability of this approach.

## Perspectives

### Selectivity

According to analysis of the analytical performance of the CD-SERS systems and their limitations, the detailed investigations of selectivity and competitive interactions are the primary directions for further studies. Importantly, besides the correct choice of the interferences (e.g., use of molecular admixtures instead of metal ions), their effect should be studied at the concentration corresponding to those in the real objects, i.e., at realistic “analyte–interfering compound” ratios. These studies are critically important for development of reliable protocols for CD-SERS analysis of the complex mixtures with deviating composition (e.g., body fluids).

### Background signal

Although a lack (or its low value) of background signal generated by native CDs was mainly reported, several reports clearly showed that the CD signal can be significant under some conditions (the “SERS signal of native CDs” section). More importantly, the conditions associated with appearing of this signal are unknown making CD-SERS assays vulnerable to unexpected factors. Therefore, detailed experimental investigation of this behavior is strongly required to improve reliability of the CD-SERS assays.

### CD-SERS sensors and electrospectral systems

As for the most of the analytical assays based on SERS, the systems discussed in this review are generally disposable (“single-shot”) assays with irreversible response. Therefore, the development of the CD-SERS sensors—CD-SERS substrates with reversible response and suitable for continuous monitoring of the analyte concentration changes in real time—is another promising direction. For example, the systems with CD-SH seem to be suitable for fabrication of the sensors accounting the strong attachment of the CD-SH and capability for realization of ratiometric signal measurements (i.e., self-calibration). Some kind of such CD-SERS system was proposed by Hill et al. for gas analysis [28] (the “Formats of CD-SERS assays” section); however, the response of the system to the concentration changes was inappropriately slow (hours). Therefore, memory effects caused by strong adsorption of the analyte molecules on the surface of SERS substrate make the development of such systems challenging and detailed fundamental studies are also required in order to overcome this limitation.

One of the directions for the development of CD-SERS sensors is fabrication of electrospectral systems based on a combination of electrochemical and SERS approaches. Reversibility of SERS response in this case can be achieved due to the regeneration of the SERS substrate surface using oxidation-reduction cycles and/or polarization-controlled sorption of analytes [63]. On the other hand, the use of inclusion complexes with CDs can improve the analytical performance due to providing additional control over redox states of the analyzed molecules, charge-transfer processes near the SERS-active surface, and mechanism of the Raman enhancement in general. Additionally, the simultaneous registration of the spectral (SERS signal) and electrochemical (*I–V* curves) information enables to get a lot of data that is favorable for discovering new effects. The fundamental studies of the CD influence on charge-transfer processes at the SERS-active surface will also be useful for better understanding of the sensing mechanism of the CD-SERS assays. For example, due to capability to stabilize anionic forms [27] and influence redox potentials [26, 27] and analyte protonation [48, 58], CDs can be tested for improving the analytical signal or inhibition of the signal of admixtures by converting them to Raman (in)active forms (reduced or oxidized). Finally, the further studies in this direction should be quite impactful if taken into account that there are only few papers on the electrospectral CD-SERS systems and that there are no analytical reports with this format analysis.

### Control measurements and critical analysis of the results

One of the most important deficiencies, which we identified during analysis of the results, is the lack of proper control measurements. For example, there are only several reports where better efficiency of the CD-SERS substrates has been proved by comparing with “standard” SERS substrates, e.g., AgNPs stabilized by citrate ions [36, 46]. Moreover, even if the control SERS substrate was used, the difference in the nanoparticle concentration for the CD-SERS and the control SERS substrates was not taken into account, which is significant lacking because intensity of SERS signal significantly depends on concentration of SERS-active material. Also, the conditions used for collection of the SERS signal from the control substrate (e.g., pH, ionic strength, the use of activation agent) were often not optimized compared to those for CD-SERS substrates. An importance of taking into account registration condition can also be seen in the work of Stiufiuc et al. [62] where 10-fold better SERS signal was obtained for CD-AuNPs compared to AgNPs. While the authors took into account that better Raman enhancement of AuNPs should be mainly connected with their aggregation (i.e., formation of “hot spots”), the use of inappropriate excitation wavelength for AgNPs (785 nm) was not considered. In the case of ratiometric CD-SERS assays, no quantitative parameters have been provided to prove superiority of this format (e.g., correlation coefficient). For the protocols where SERS substrate was prepared by reduction and stabilization of metal nanoparticles using pure CDs or its mixture with glucose, the fate and effect of the byproducts were not investigated (e.g., products of oxidation of CDs or glucose). Finally, preliminary demonstration that the CD of a choice can form an inclusion complex with the analyte of interest and investigation of this inclusion complex will provide additional control over the assay performance and reliability. Therefore, detailed planning of the control experiments in the further studies significantly improves the reliability of the results.

### Use of available information on CD inclusion complexes

As described in the “Introduction” section, inclusion complexes with CDs have been used for solubilization and stabilization of various molecules in food science [13] and pharmacy [12], for purification of water [14], and for separation and chiral recognition in analytical chemistry [15–17]. Thus, there is a huge value of information about the CD-based inclusion complexes; however, it is surprising that only well-known affinity of CDs toward polyaromatic hydrocarbons and polychlorinated biphenyls have been mainly used for the development of CD-SERS assays. Besides enabling maximization of adsorption efficiency and the signal intensity, such information can be extremely useful in chemical analysis for predicting and overcoming competitive interactions with interfering components. Also, such information facilitates the choice of a more suitable CD (α-, β-, or γ-), its derivative (with (non-)polar, charged or neutral moieties; single CD or CD-modified polymer), and its concentration (“analyte–CD” ratio). Finally, in the case of fundamental studies, additional information about the structure of the CD inclusion complexes obtained by different methods can be very helpful for the explanation of Raman enhancement mechanism and the effect of CDs on it.

## Conclusions

Summing up findings of this review, we can conclude that the CD-SERS systems are attractive for further applications in SERS-based chemical analysis and solution of analytical challenges. This is possible due to excellent cost-efficiency, availability, and well-known properties of CDs as well as availability of reference data about the CD inclusion complexes. The diversity of the analysis formats based on CD-SERS systems also supports flexibility of the CD-SERS analysis. Additionally, SERS studies of the CD inclusion complexes can improve understanding of the Raman enhancement mechanisms. However, while having useful properties, CDs also have limitations (e.g., limited selectivity and moderate binding with analytes) and the significant attention in the further studies should be paid to proper estimation of these limitations and development of approaches to overcome them. Therefore, we hope that this review will be a fruitful source of the ideas for further fundamental and application studies and will speed up the progress in this direction.
